# Ubiquinol (reduced Coenzyme Q10) in patients with severe sepsis or septic shock: a randomized, double-blind, placebo-controlled, pilot trial

**DOI:** 10.1186/s13054-015-0989-3

**Published:** 2015-07-01

**Authors:** Michael W. Donnino, Sharri J. Mortensen, Lars W. Andersen, Maureen Chase, Katherine M. Berg, Julia Balkema, Jeejabai Radhakrishnan, Raúl J. Gazmuri, Xiaowen Liu, Michael N. Cocchi

**Affiliations:** Department of Emergency Medicine, Beth Israel Deaconess Medical Center, One Deaconess Road West CC-2, Boston, MA 02215 USA; Department of Medicine, Division of Pulmonary and Critical Care, Beth Israel Deaconess Medical Center, One Deaconess Road West CC-2, Boston, MA USA; Research Center for Emergency Medicine, Aarhus University Hospital, Norrebrogade 44, Aarhus, 8000 Denmark; Department of Anesthesiology, Aarhus University Hospital, Norrebrogade 44, Aarhus, 8000 Denmark; Resuscitation Institute at Rosalind Franklin University of Medicine and Science, 3333 Green Bay Road, North Chicago, IL 60064 USA; Department of Anesthesia Critical Care, Division of Critical Care, Beth Israel Deaconess Medical Center, One Deaconess Road West CC-2, Boston, MA USA

## Abstract

**Introduction:**

We previously found decreased levels of Coenzyme Q10 (CoQ10) in patients with septic shock. The objective of the current study was to assess whether the provision of exogenous ubiquinol (the reduced form of CoQ10) could increase plasma CoQ10 levels and improve mitochondrial function.

**Methods:**

We performed a randomized, double-blind, pilot trial at a single, tertiary care hospital. Adults (age ≥18 years) with severe sepsis or septic shock between November 2012 and January 2014 were included. Patients received 200 mg enteral ubiquinol or placebo twice a day for up to seven days. Blood draws were obtained at baseline (0 h), 12, 24, 48, and 72 h. The primary outcome of the study was change in plasma CoQ10 parameters (total CoQ10 levels, CoQ10 levels relative to cholesterol levels, and levels of oxidized and reduced CoQ10). Secondary outcomes included assessment of: 1) vascular endothelial biomarkers, 2) inflammatory biomarkers, 3) biomarkers related to mitochondrial injury including cytochrome *c* levels, and 4) clinical outcomes. CoQ10 levels and biomarkers were compared between groups using repeated measures models.

**Results:**

We enrolled 38 patients: 19 in the CoQ10 group and 19 in the placebo group. The mean patient age was 62 ± 16 years and 47 % were female. Baseline characteristics and CoQ10 levels were similar for both groups. There was a significant increase in total CoQ10 levels, CoQ10 levels relative to cholesterol levels, and levels of oxidized and reduced CoQ10 in the ubiquinol group compared to the placebo group. We found no difference between the two groups in any of the secondary outcomes.

**Conclusions:**

In this pilot trial we showed that plasma CoQ10 levels could be increased in patients with severe sepsis or septic shock, with the administration of oral ubiquinol. Further research is needed to address whether ubiquinol administration can result in improved clinical outcomes in this patient population.

**Trial registration:**

Clinicaltrials.gov identifier NCT01948063. Registered on 18 February 2013.

## Introduction

Severe sepsis and septic shock continue to be leading causes of morbidity and mortality despite widespread implementation of strategies focused on early antibiotic administration and aggressive resuscitation [1]. Annually there are an estimated 751,000 cases of severe sepsis in the United States, with mortality upwards of 29 % [2]. Sepsis-induced multiple organ system failure is a common final pathway believed to be caused by a systemic inflammatory response involving micro- and macro-circulatory dysfunction and alterations in cellular metabolism, particularly mitochondrial dysfunction, though the exact mechanisms remain unknown [3–5]. Recent large trials of specific hemodynamic interventions have failed to show a clear benefit in patients with severe sepsis or septic shock [6–8], and new adjunct therapies are needed to improve outcomes.

Coenzyme Q10 (CoQ10) is a fat-soluble molecule synthesized in the mitochondrial inner membrane that exists both in oxidized form (ubiquinone) and reduced form (ubiquinol). The mitochondrial electron transport chain contains two transporters (CoQ10 and cytochrome *c*) and four complexes (I-IV). CoQ10 plays an essential role in the electron transport chain as the carrier of electrons from complex I and II to complex III. Disruption of this mechanism can compromise oxidative phosphorylation, thereby leading to decreased levels of cellular energy (adenosine triphosphate (ATP)) production [9].

Our group has previously found decreased levels of CoQ10 in patients with septic shock [10], indicating the possibility that cellular energy production is compromised in this disease state. We also found that levels of CoQ10 were inversely associated with levels of vascular cell adhesion molecule 1 (VCAM-1) and the anti-inflammatory cytokine interleukin-10 (IL-10), suggesting that CoQ10 may also play a role in vascular endothelial dysfunction and the inflammatory response seen in severe sepsis and septic shock [10].

Based on these findings, we hypothesized that CoQ10 deficiency in septic patients may contribute to mitochondrial dysfunction, and that this dysfunction could be mitigated by exogenous administration of ubiquinol (reduced CoQ10). Thus, we conducted a randomized, double-blind, placebo-controlled, pilot trial providing enteral ubiquinol supplementation to patients with severe sepsis or septic shock to determine absorption and the effect of supplementation on systemic levels, as well as the effect of ubiquinol on mitochondrial injury, markers of inflammation, and markers of vascular endothelial injury.

## Materials and methods

### Design and setting

This was a single-center, randomized, double-blind, pilot phase II trial comparing ubiquinol to placebo in patients with severe sepsis or septic shock. The study was conducted at Beth Israel Deaconess Medical Center (BIDMC), which is an urban tertiary care hospital in Boston, Massachusetts with approximately 55,000 emergency department visits annually and a total of 77 intensive care unit beds. The study was approved by the Committee on Clinical Investigations at BIDMC (approval number: 2012P-000267) and patients or their legally authorized surrogate provided written informed consent prior to enrollment. The trial was registered at Clinicaltrials.gov (identifier: NCT01948063) and was sponsored by Kaneka Corporation, Japan. The trial was investigator-initiated and the sponsor was not involved in study design or conduct, and had no role in manuscript preparation.

### Study population

The hospital’s emergency department and intensive care units were screened for eligible patients between November 2012 and January 2014. Inclusion criteria were that patients were aged ≥18 years and were diagnosed with severe sepsis or septic shock. Severe sepsis was defined as the presence of two or more systemic inflammatory response syndrome criteria, documented or suspected infection, and evidence of sepsis-induced organ dysfunction or tissue hypoperfusion as defined by the Surviving Sepsis campaign [1]. Septic shock was defined as sepsis-induced hypotension persisting despite adequate fluid resuscitation, in accordance with the Surviving Sepsis campaign definitions [1].

We excluded patients based on the following criteria: 1) currently on CoQ10 supplementation, 2) unable to receive enteral medication per the clinical team, 3) enrolled in another ongoing study, 4) non-English speaking, 5) patient unable to consent and legally authorized surrogate not present, 6) ‘do not resuscitate’, ‘do not intubate’ or ‘comfort measures only’ designation, or 7) member of a protected population.

### Randomization, study drug, and blinding

A master randomization list was created using SAS (SAS Institute, Cary, NC, USA), randomizing patients to ubiquinol or placebo in a 1:1 ratio. The list was located in the central pharmacy for the duration of the study. The study drug (placebo or ubiquinol) was administered as a pill by mouth or in liquid form per nasogastric tube if present. Given the distinct taste of the liquid, this was not provided to all patients. The study drug was administered twice per day after enrollment, and was continued daily for seven days or until hospital discharge, whichever came first. Ubiquinol (Kaneka Corporation, Japan) was administered at a dosage of 200 mg per dose. Ubiquinol was chosen over ubiquinone (oxidized CoQ10) due to its higher bioavailability [11]. The dose was chosen based on safety data showing that doses of 300 mg are well-tolerated in healthy volunteers [11], and the dose was comparable to that used in a previous post-cardiac arrest trial [12]. The placebo was in identical pills or liquids as the study drug and patients, healthcare personnel, and the research team remained blinded throughout the study period.

### Blood samples and data collection

Venous blood was collected at enrollment immediately before administration of study drug (0 h) and at 12, 24, 48, and 72 h thereafter. Samples were centrifuged and serum and plasma were aliquoted into light-protected cryotubes and immediately frozen at −80 °C.

Upon enrollment, we recorded demographic data, co-morbid conditions, vital signs, clinical laboratory values, whether or not the patient was receiving mechanical ventilation or vasopressors, and calculated the Acute Physiology and Chronic Health Evaluation II (APACHE II) score. Vital signs and laboratory values were also collected at all subsequent time points and outcome data, including length of intensive care unit stay, length of hospital stay, and mortality, was collected at patient discharge. All data was entered into a secure, online database by trained research assistants.

### Outcome measures

The primary outcome of the study was change in plasma CoQ10 parameters (total CoQ10 levels, CoQ10 levels relative to cholesterol levels, levels of oxidized (ubiquinone) and reduced (ubiquinol) CoQ10, and the fraction of reduced CoQ10 (reduced CoQ10/total CoQ10 levels)).

Secondary outcomes included were: 1) assessment of vascular endothelial function via vascular endothelial growth factor (VEGF) and VCAM-1 measurements, 2) assessment of inflammation via IL-2, IL-6, IL-10, and tumor necrosis factor (TNF)-α measurements, 3) assessment of mitochondrial injury via cytochrome *c* levels and DNA markers, and 4) assessment of clinical outcomes (length of ICU and hospital stay, and in-hospital mortality).

### Measurement of Coenzyme Q10 levels and biomarkers

CoQ10 levels were measured in plasma samples by the Department of Pathology and Laboratory Medicine at Cincinnati Children’s Hospital Medical Center (Cincinnati, Ohio, USA) using high-performance liquid chromatography as previously described in detail [13]. We measured three different CoQ10 parameters (total CoQ10, oxidized CoQ10, and reduced CoQ10 (all measured in μg/mL)), as well as total cholesterol levels. From these, two additional parameters were calculated: total CoQ10/cholesterol and reduced CoQ10/total CoQ10. CoQ10 levels were measured at the 0, 12, and 24 h time points.

Plasma samples were analyzed for multiple vascular endothelial and inflammatory markers (VEGF, VCAM-1, IL-2, IL-6, IL-10, and TNF-α) using customized Meso Scale Discovery Human Multiplex Panel (Rockville, Maryland, USA). All samples were measured in duplicate with the inter-assay coefficients of variability ranging from 2.2 to 5.8 %. VCAM-1 is reported in log-transformed μg/mL and the rest of the markers in log-transformed pg/mL. Cytokine levels were measured at the 0, 12, 24, 48 and 72 h time points.

Cytochrome *c* levels were measured by electrochemiluminescence in a Quickplex SQ 120 instrument (Meso Scale Discovery) at the Resuscitation Institute/Rosalind Franklin University of Medicine and Science (North Chicago, IL, USA). Cytochrome *c* levels had previously been reported to correlate inversely with survival after cardiac arrest in rodents [14]. Standard curves were prepared using commercially available human heart cytochrome *c* (cat# C3483-10UG, Sigma-Aldrich, St. Louis, MO, USA) diluted in 10 % pooled human plasma from healthy volunteers in assay buffer at concentrations of 1,200.0 ng/mL, 400.0 ng/mL, 133.3 ng/mL, 44.4 ng/mL, 14.8 ng/mL, 4.9 ng/mL, 1.6 ng/mL, and 0 ng/mL. Each plasma sample with an unknown cytochrome *c* concentration was thawed in ice and diluted 1:1 in the assay buffer. Samples and standards (in a volume of 25 μL) were run in duplicate. Data analysis was performed using Meso Scale Discovery software, which developed the standard curve used to calculate the unknown concentrations. The upper limit of detection was 1,200 ng/mL and the lower limit of detection was 4 ng/mL, which is defined as 2.5 standard deviations above the background mean. The software also calculates the coefficient of variation between the replicate samples. If the sample cytochrome *c* concentration fell above or below the dynamic range of the standard curve, the assay was repeated after appropriate dilution. Cytochrome *c* levels were expressed in log-transformed ng/mL. Cytochrome *c* levels were measured at the 0, 12, and 24 h time points. Cytochrome *c* was included as an exploratory outcome as a potential marker of mitochondrial injury [14].

We measured cell-free DNA, two markers of nuclear DNA, and two markers of mitochondrial DNA (mtDNA) at the 0, 12, and 24 h time points. In order to measure cell-free DNA in plasma, the plasma samples were centrifuged at 16,000 g for 10 min to remove any residual cells. The upper portion of the plasma was removed into a nuclease-free tube and stored at −80 °C prior to DNA extraction. Cell-free DNA in the plasma was isolated using a plasma/serum DNA isolation kit (Abcam, Cambridge, MA, USA) according to the manufacturer’s protocol. The amount of cell-free DNA was measured using a NanoDrop 1000 Spectrophotometer (Thermo Fisher Scientific, Wilmington, DE, USA). The quality of DNA was checked by the A260/280 ratio. The amount of mtDNA was measured with *mtDNA-tRNA*^*leu*^ and *mtDNA-D* loop genes, which are specifically present in the mitochondrial genome. Nuclear DNA was amplified using the single-copy beta-2-microglobulin (*B2M*) and *RNase P* nuclear genes. Primers and probes for real-time PCR were purchased from Life Technologies Corporation and utilized as previously described by Bai and Wong [15]. TaqMan real-time quantitative PCR assay was performed on a 7500 real time PCR system following the manufacturer’s protocol (Applied Biosystems, Life Technologies Corporation, CA, USA). DNA extracted from whole blood with a two-fold serial dilution was used to construct the calibration curve. All samples were measured in duplicate with the inter-assay coefficients of variability ranging from 1.6 to 5.5 %. Cell-free DNA was measured as a marker of cellular injury, as well as a surrogate for poor outcome [16–18].

### Statistical analyses

Descriptive statistics were used to summarize the study population. Data for continuous variables are presented as means with standard deviations (SD) or medians with quartiles, depending on the normality of the data. Categorical data are presented as counts with frequencies. Depending on the distribution of the data, t-tests or Wilcoxon rank sum tests were used to compare continuous data between the groups. Categorical data were compared using chi-squared or Fisher’s exact tests as appropriate.

To assess the difference in repeated continuous variables (CoQ10 levels, vascular and inflammatory biomarkers, cytochrome *c* levels, and DNA markers) between groups we used repeated measures models to account for within-subject correlation. Variables were log-transformed before statistical analysis. An appropriate covariance structure was chosen and the residuals were visually assessed for normality. Due to the exploratory nature of the current study we did not account for multiple comparisons. Statistical analyses were conducted with the use of SAS software, version 9.3 (SAS Institute Inc., Cary, NC, USA). All hypothesis tests were two-sided, with a significance level of *P* <0.05.

## Results

### Study population

**Fig. 1:**
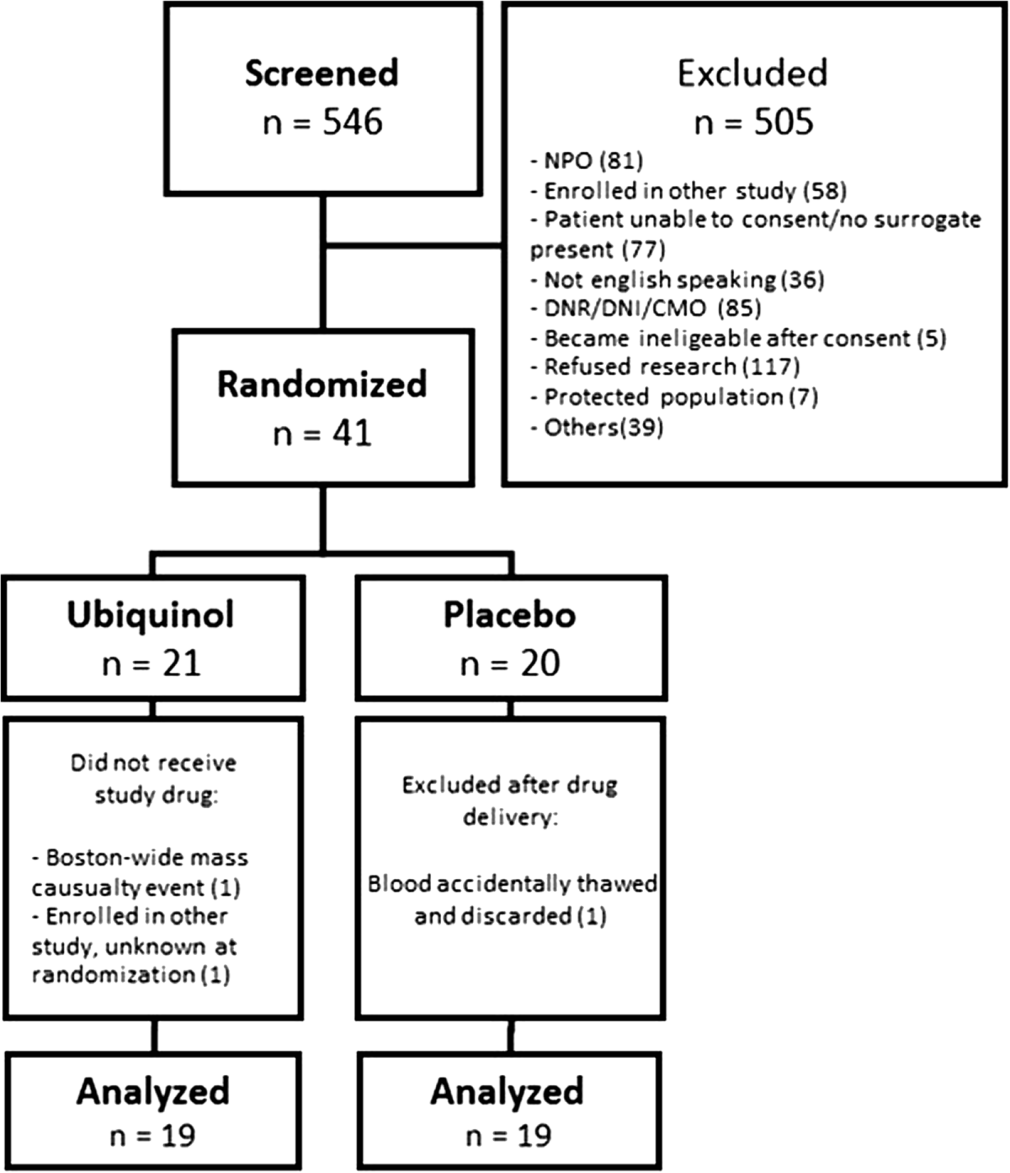
Consolidated Standards for Reporting Trials (CONSORT) diagram. A total of 546 patients were screened. Of these 41 were randomized and 38 were analyzed. CMO: Comfort measures only, DNI: Do Not Intubate, DNR: Do Not Resuscitate NPO: nil per os

A total of 546 patients with severe sepsis or septic shock were screened and 41 patients were randomized. Of those, three were excluded post-randomization (two in the ubiquinol group and one in the placebo group), leaving 38 for the final analysis (see Fig. 1). The median age was 62 ± 16 years and 47 % were female. The overall mortality was 16 %. There were no statistically significant differences between the baseline characteristics for the ubiquinol group and the placebo group, as illustrated in Table 1.

**Table 1:** Selected baseline characteristics^a^

Characteristic	Ubiquinol (*n* = 19)	Placebo (*n* = 19)
Demographics		
Age (years)	60 ± 18	64 ± 14
Sex (female)	11 (58 %)	7 (37 %)
Race (white)	14 (78 %)	15 (83 %)
Co-morbidities		
Coronary artery disease	2 (11 %)	2 (11 %)
Congestive heart failure	2 (11 %)	2 (11 %)
Hyperlipidemia	6 (32 %)	2 (11 %)
Hypertension	12 (63 %)	9 (47 %)
Chronic obstructive pulmonary disease	2 (11 %)	3 (16 %)
Diabetes	6 (32 %)	8 (42 %)
Liver disease	1 (6 %)	0 (0)
Renal disease	3 (16 %)	1 (6 %)
Cancer	4 (21 %)	9 (47 %)
Vital signs at enrollment		
Heart rate	92 ± 19	88 ± 22
Systolic blood pressure	109 ± 19	105 ± 11
Diastolic blood pressure	54 ± 16	61 ± 11
Respiratory rate	25 ± 6	22 ± 6
Temperature	98.4 ± 1.4	98.7 ± 1.1
Laboratory values at enrollment		
Lactate (mmol/L)	1.6 (1.1-2.5)	1.3 (1.0-1.6)
Creatinine (mg/dL)	1.7 (1.0-3.0)	1.0 (0.7-1.7)
Bicarbonate (mEq/L)	30 ± 7	34 ± 7
White blood count (K/μL)	13.2 ± 6.3	16.5 ± 11.4
Hematocrit (%)	30.0 ± 7.0	33.7 ± 7.4
Source of sepsis		
Pneumonia	9 (47)	7 (37)
Urinary tract	2 (11)	2 (11)
Intraabdominal	1 (5)	3 (16)
Skin, joint, or soft tissue	2 (11)	4 (21)
Endocarditis	1 (5)	0 (0)
Unclear	4 (21)	3 (16)
Positive blood culture	5 (26)	6 (32)
Mechanical Ventilation	11 (58 %)	7 (37 %)
Vasopressors	12 (63 %)	12 (63 %)
APACHE II score at enrollment	19 ± 9	18 ± 10
Baseline CoQ10 Levels		
Total CoQ10 (mcg/mL)	0.37 (0.30-0.62)	0.33 (0.25-0.69)
CoQ10 Reduced (mcg/mL)	0.31 (0.20-0.40)	0.30 (0.22-0.66)
CoQ10 Oxidized (mcg/mL)	0.05 (0.02-0.15)	0.03 (0.02-0.04)
CoQ10 %Reduced	0.87 (0.73-0.94)	0.92 (0.90-0.95)
CoQ10: Cholesterol	0.38 (0.28-0.65)	0.36 (0.26-0.55)

^a^Baseline values with continuous values expressed as means ± standard deviation or median (quartiles), depending on normality of the data. Categorical values are counts with frequencies. No comparisons had a *P* value <0.05

### Coenzyme Q10 levels

**Fig. 2:**
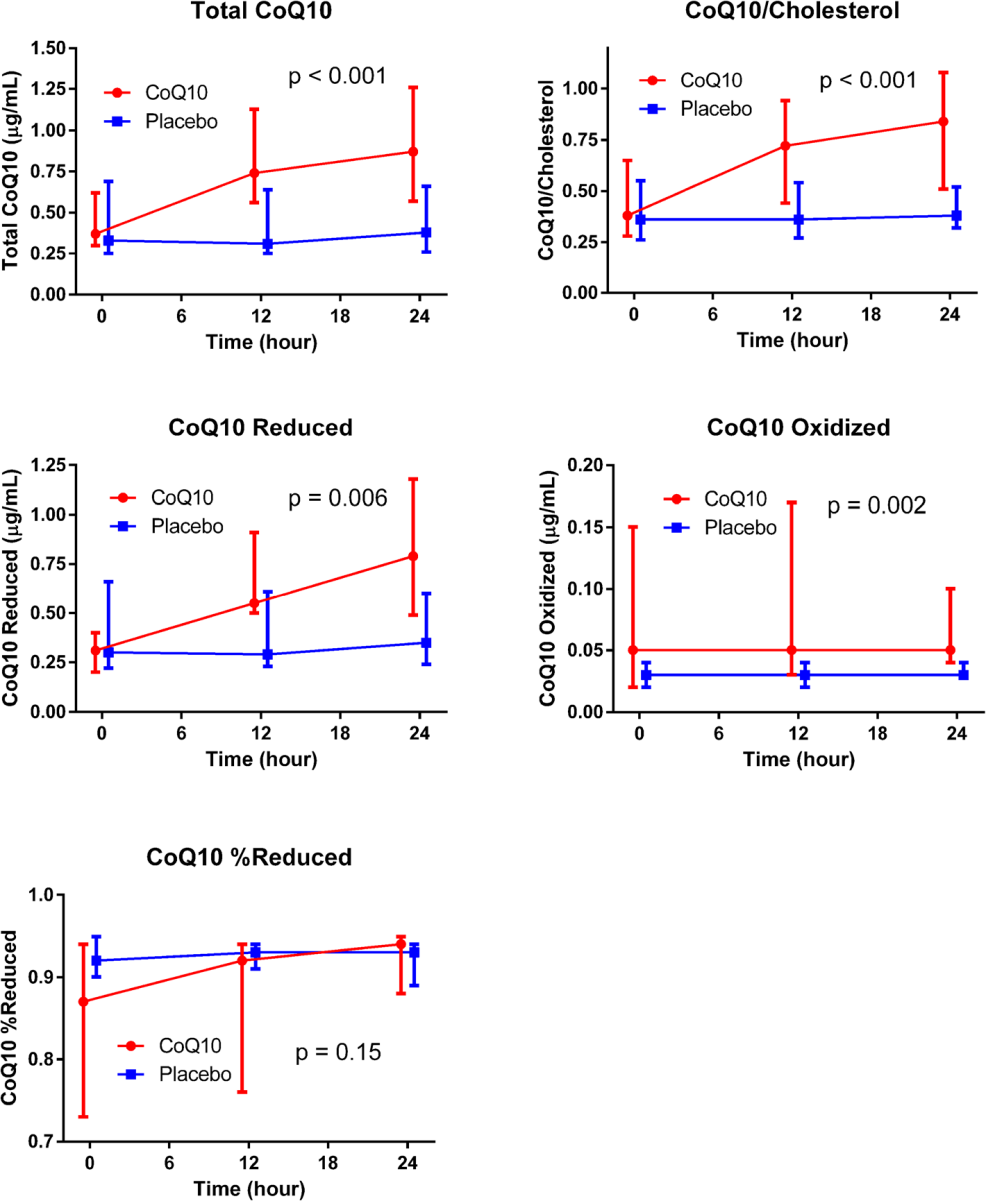
CoQ10 parameters over time. Values are reported as medians with errors bars indicating first and third quartiles. The *P* values represent the global tests that values are similar between groups at the 12 and 24 h time points

The baseline CoQ10 levels in the two groups were similar. At 12 and 24 h after study drug administration there were increased levels of total CoQ10 (*P* <0.001), CoQ10 levels relative to cholesterol levels (*P* <0.001), reduced CoQ10 levels (*P* = 0.006) and oxidized CoQ10 levels (*P* = 0.002) in the ubiquinol-treated group compared to the placebo group. There was no statistically significant difference in the fraction of reduced CoQ10 (*P* = 0.15) (Fig. 2).

### Vascular endothelial and inflammatory markers

**Fig. 3:**
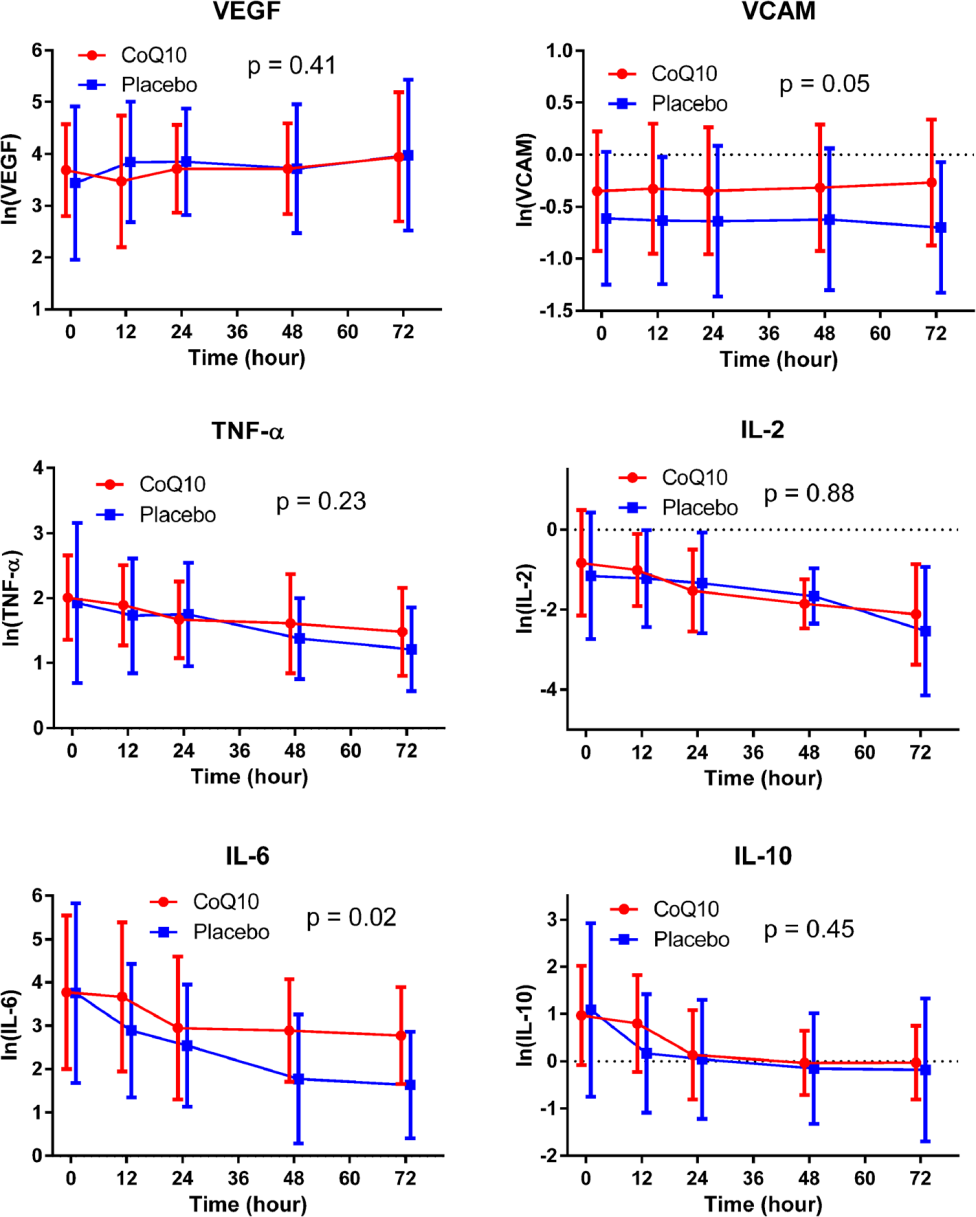
Vascular and inflammatory biomarkers over time. Values are reported as log-transformed means with errors bars indicating standard deviations. The *P* values represent the global tests that values are similar between groups at the 12, 24, 48, and 72 h time points

There was no difference in baseline vascular endothelial and inflammatory markers between the groups. IL-6 levels at 12, 24, 48, and 72 h were higher in patients receiving ubiquinol as compared to those receiving placebo (*P* = 0.02). There was no difference between the ubiquinol and placebo groups in levels of VEGF (*P* = 0.41), VCAM-1 (*P* = 0.05), TNF-α (*P* = 0.23), IL-2 (*P* = 0.88), or IL-10 (*P* = 0.45) over time (Fig. 3).

### Cytochrome *c*

**Fig. 4:**
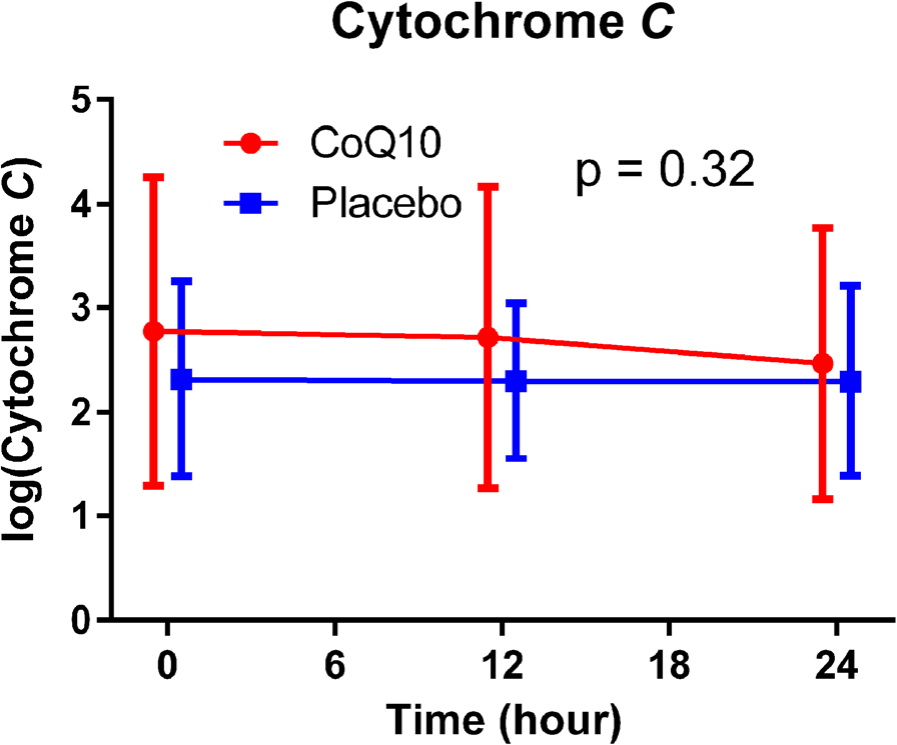
Cytochrome *c* values over time. Values are reported as log-transformed means with errors bars indicating standard deviations. The *P* values represent the global tests that values are similar between groups at the 12 and 24 h time points

There was no difference in baseline cytochrome *c* levels between groups. At 12 and 24 h after study drug administration, there was also no difference in cytochrome *c* levels between groups (*P* = 0.32, Fig. 4).

### Nuclear and mitochondrial DNA

**Fig. 5:**
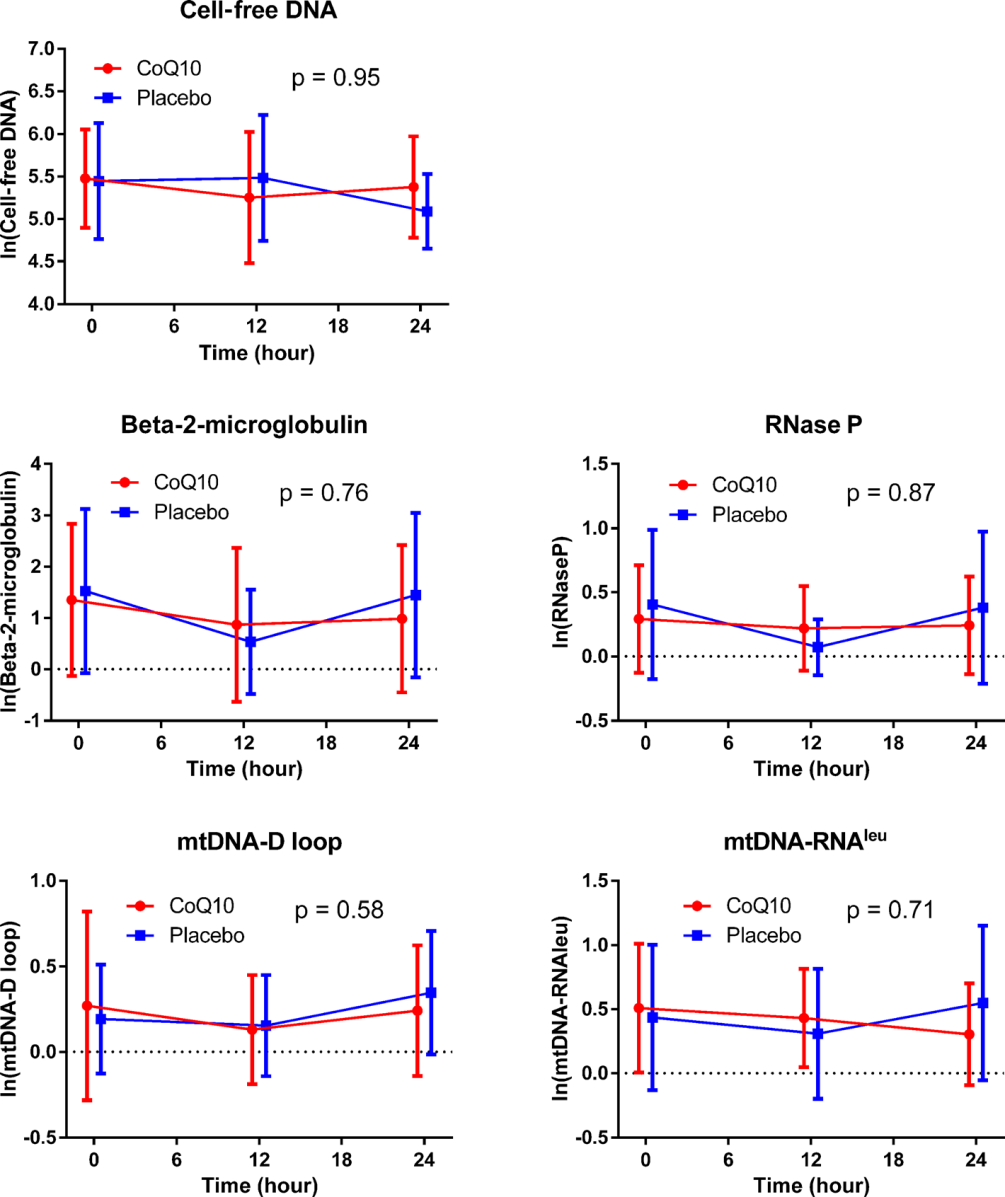
Cell-free DNA, and nucleus and mitochondrial DNA values over time. Values are reported as log-transformed means with errors bars indicating standard deviations. The *P* values represent the global tests with values that are similar between groups at the 12 and 24 h time points

There was no difference in baseline cell-free DNA, *B2M*, *RNase P*, *mtDNA-D* loop, or *mtDNA-RNA*^*leu*^ between the groups. There was no difference in cell-free DNA between the two groups (*P* = 0.95). There was no difference in the fold change of *B2M* (*P* = 0.76), *RNase P* (*P* = 0.87), *mtDNA-D* loop (*P* = 0.58), or *mtDNA-RNA*^*leu*^ (*P* = 0.71) over time (Fig. 5).

### Clinical outcomes

There was no difference in ICU length of stay (5 (3 to 7) versus 3 (2 to 6) days, *P* = 0.30) or hospital length of stay (11 (6 to 19) days versus 10 (6 to 19) days, *P* = 0.82) between the two groups. These results were similar when only analyzing survivors. There was no difference in in-hospital mortality between patients receiving ubiquinol versus placebo (4 (21 %) versus 2 (11 %), *P* = 0.66). The four deaths in the ubiquinol group happened 1, 1, 5, and 16 days after the drug administration. The mode of death was determined to be comorbid withdrawal of care in two patients with underlying terminal illness, refractory hypotension in one patient with underlying terminal illness, and progressive respiratory failure in one patient with underlying alcohol-associated hepatic failure. The mode of death for the two patients in the placebo group was determined as comorbid withdrawal of care in one patient with terminal illness, and refractory hypotension in one patient with underlying terminal illness (both deaths occurred four days after drug administration). As noted, the majority of deaths occurred in patients with a preexisting terminal illness, including metastatic cancer or end-stage organ failure. None of the deaths were determined to be related to the study medication as assessed by an independent data safety monitoring board. Gastrointestinal disturbance, potentially related to ubiquinol administration, was reported in one patient.

## Discussion

In this pilot study of ubiquinol versus placebo in patients with severe sepsis and septic shock, plasma CoQ10 levels increased in patients who received ubiquinol. We found statistically significant higher levels of IL-6 in patients receiving ubiquinol as compared to those receiving placebo. The reason for this finding remains largely unexplained, but could be related to multiple testing. The assessment of other biomarkers related to vascular endothelial function, mitochondrial dysfunction, and inflammation revealed no statistically significant difference between the ubiquinol and the placebo groups in this study.

CoQ10, as a key component of the electron transport chain, plays an important role in the mitigation of oxidative stress and production of cellular energy [19]. Previous investigations have demonstrated that CoQ10 levels are low in critical illness, specifically septic shock and post-cardiac arrest [10, 20, 21]. Multiple rat models have been used to test and demonstrate the beneficial impact of CoQ10 in sepsis and have found decreased oxidative stress, prevention of mitochondrial damage, less renal and acute liver dysfunction, and neuroprotective effects [22–24]. In the current study, we demonstrate that exogenous provision of ubiquinol increases CoQ10 levels in patients with severe sepsis and septic shock. The potential benefits of this increase remain unclear.

Severe sepsis is associated with respiratory, cardiovascular, and renal dysfunction, with significant alterations to metabolic pathways and cellular function [25, 26]. A central hypothesis in organ dysfunction is the concept of tissue hypoxia [27]. Cytopathic hypoxia (compromised cellular oxygen utilization), rather than inadequate oxygen delivery, may play a significant role in the development of multi-organ dysfunction and lactate elevation in sepsis [28, 29], although the exact mechanisms remain unknown [30]. Despite optimization of hemodynamics and oxygen therapy, oxygen may not be utilized at the mitochondrial level. Mitochondria consume 90 % of cellular oxygen in order to generate energy as ATP, via oxidative phosphorylation by transfer of electrons from the Krebs cycle to the electron transport chain, via nicotinamide adenine dinucleotide and 5,10-methylenetetrahydrofolate reductase [31]. The electron transport chain is located on the inner mitochondrial membrane and consists of four complexes and two transporters. CoQ10 plays an essential role in the electron transport chain as the carrier of electrons from complex I and II to complex III. Disruption of this mechanism can compromise oxidative phosphorylation in the mitochondria, thereby leading to decreased levels of cellular ATP production [9].

Previous studies have demonstrated a beneficial role of CoQ10 as a co-adjutant in the treatment of syndromes characterized by impaired mitochondrial bioenergetics and increased oxidative stress [32]. CoQ10 has shown beneficial effects on diastolic and systolic function in patients with chronic heart failure [33–35] and, in a single-center pilot trial, CoQ10 was found to increase three-month survival when given to post-cardiac arrest patients [12]. Moreover CoQ10 has shown promising clinical benefits in small human studies of progressive supranuclear palsy [36], Huntington’s disease [37], and Friedreich’s ataxia [38]. Finally, CoQ10 has also been studied in Parkinson’s disease, with inconsistent results [39–41].

In the current study we were unable to detect any differences in secondary endpoints between the groups. There are multiple possible explanations for this lack of effect. Our sample size was relatively small, and we may have been underpowered to detect clinically relevant differences in biomarkers or clinical outcomes. A relatively large portion of potentially eligible patients were excluded, which may decrease the generalizability of our findings. Given the lack of human trials in septic patients, we used a relatively low dose (200 mg twice per day). Previous studies have demonstrated that oral consumption of CoQ10 at dosages of 400, 600, 800, and 1,200 mg per day are well tolerated in patients with Parkinson’s disease, with the greatest benefit in the group receiving the highest dosage (1,200 mg per day) [40, 39]. As such, our dose may have been inadequate to show a treatment effect, and higher doses of ubiquinol may be required to compensate for the severity of metabolic dysfunction and energy depletion that occurs in severe sepsis and septic shock.

The timing and duration of our intervention may have influenced the results. Approximately half of our patients were mechanically ventilated and comatose at the time of enrollment, which led to some delay in the administration of the first dose as consent had to be obtained from a legally authorized surrogate. The duration of therapy was relatively short (maximum of seven days), and a longer duration and follow-up period may be needed, especially for long-term patient-centered outcomes. For example, ubiquinol could theoretically protect against cognitive dysfunction or muscular weakness post-septic shock, but we did not measure these longer-term outcomes. Further, the majority of our biomarker endpoints were only assessed within the first 24 h. It is possible that changes could have been seen if the endpoints were evaluated at later time points. The enrolled patient population was heterogeneous, with a relatively low overall mortality (16 %) that, based on review by a blinded physician, was mainly related to withdrawal of care in patients with underlying terminal illness. Future studies might consider excluding patients with terminal illness. Whether certain subgroups, such as a more acutely ill population with potential signs of mitochondrial injury (for example, elevated lactate), may benefit remains unknown. Lastly, the possibility remains that ubiquinol is ineffective as a ‘metabolic resuscitator’ in patients with severe sepsis and septic shock. However, additional studies are needed before any firm conclusions can be made.

## Conclusions

We have shown that ubiquinol is absorbed in patients with severe sepsis and septic shock. There were no significant changes in VEGF, VCAM-1, the majority of inflammatory markers, or clinical outcomes, such as mortality and length of ICU and hospital stay. Further investigation with different dosages, timing of drug delivery, or more specific populations may be warranted to determine whether ubiquinol administration may be beneficial as an adjunct therapy in patients with severe sepsis and septic shock.

## Key messages

- Coenzyme Q10 is an essential component of the mitochondrial electron transport chain.
- In this placebo-controlled, randomized, pilot trial we found that enteral administration of ubiquinol (reduced coenzyme Q10) increases total Coenzyme Q10 levels, Coenzyme Q10 levels relative to cholesterol levels, and levels of oxidized and reduced Coenzyme Q10.
- There was no difference in vascular endothelial biomarkers, inflammatory biomarkers, or biomarkers related to mitochondrial injury, including cytochrome c levels.
- We found no difference in clinical outcomes between the ubiquinol and placebo groups.

## Abbreviations

APACHE II: Acute Physiology and Chronic Health Evaluation II
ATP: Adenosine triphosphate
B2M: Beta-2-microglobulin
BIDMC: Beth Israel Deaconess Medical Center
CoQ10: Coenzyme Q10
ICU: Intensive care unit
IL: Interleukin
mtDNA: Mitochondrial DNA
SD: Standard deviations
TNF: Tumor necrosis factor
VCAM-1: Vascular cell adhesion molecule
VEGF: Vascular endothelial growth factor
